# The Bidirectional Interplay Between Substances of Abuse and Gut Microbiome Homeostasis

**DOI:** 10.3390/life15060834

**Published:** 2025-05-22

**Authors:** Alejandro Borrego-Ruiz, Juan J. Borrego

**Affiliations:** 1 Departamento de Psicología Social y de las Organizaciones, Universidad Nacional de Educación a Distancia (UNED), 28040 Madrid, Spain; 2 Departamento de Microbiología, Universidad de Málaga, 29071 Málaga, Spain; jjborrego@uma.es

**Keywords:** human gut microbiome, bacterial dysbiosis, drugs of abuse, substance use disorders, clinical studies

## Abstract

Specific gut microorganisms and their metabolic by-products have been identified as key regulators of host physiology, contributing to the modulation of the immune system, inflammatory processes, brain function, and behavior, which highlights the gut microbiome as a potential modulator of the neurobiological mechanisms involved in substance use disorders. This narrative review provides an updated overview of how drugs of abuse influence the composition and dynamics of the human gut microbiome and how bacterial dysbiosis may be a contributing factor to substance use disorders by modulating the communication between the gut and the brain. Thus, by examining commonly abused substances such as alcohol, psychostimulants, opioids, cannabinoids, and nicotine, this review aimed to deepen the understanding of the bidirectional relationship between the gut microbiome and substance use. There is evidence indicating that gut microbiome alterations may influence addiction through changes in gut-brain signaling. Furthermore, changes in the gut microbiome and its metabolites may not only result from substance use disorders, but could also modulate behavioral responses to drugs of abuse. Although the exact mechanisms by which the gut microbiome modulates behavioral responses to drugs of abuse are not fully understood, microbial products such as short-chain fatty acids, tryptophan metabolites, bile acids, and neurotransmitters have been suggested to play a role in this process by influencing the blood–brain barrier permeability, host immune activation, neural signaling, and gene expression. Therefore, manipulating the gut microbiome or its by-products may represent a promising approach for enhancing substance use disorder treatments, identifying individuals at increased risk of pathological drug use, and elucidating its role in substance-related behaviors.

## 1. Introduction

Initial experimentation with drugs of abuse is typically prompted by curiosity and expansion motives, but through repeated reinforcement, this behavior can transform into a habitual pattern, ultimately culminating in a substance use disorder (SUD) [1]. SUDs are psychiatric conditions with a high morbidity worldwide, characterized by the abusive and/or hazardous consumption of one or multiple drugs [2]. Although SUDs can include several substances with different molecular mechanisms of action, in most cases, they share common patterns such as craving, seeking, dependence, abstinence, and relapse [2]. SUDs result in functional and health-related adverse outcomes, constituting a significant threat to individual and community well-being [3]. Evidence-based treatments can support recovery, but evolving substance use patterns challenge their long-term effectiveness, highlighting the need for new approaches to address factors such as drug potency, overdose risk, and the emergence of new substances and methods of use [3]. In addition, an increase in consumption intensity and more severe SUDs among young populations further underscores the need to understand the underlying mechanisms involved in substance use [4].

Substance use exerts a direct neurobiological effect and triggers different behavioral responses that can be directly or indirectly modulated by various host systems such as the endocrine system [5,6], the immune system [7], and the gut microbiome (GM) [8,9]. A multitude of mechanisms and pathways have been postulated through which SUDs can influence the diversity and composition of the GM including dietary habits, xenobiotic effects of the drugs, and alteration of the neural regulation of gut motility [10,11,12,13].

The human GM harbors a highly diverse and dense microbial community, estimated to contain between 10^11^ and 10^12^ microbial cells per milliliter [14]. This community encompasses representatives from multiple microbial domains, including bacteria, archaea, protozoa, viruses, and fungi, with bacteria constituting the most predominant taxon [15,16]. The bacteria domain comprises more than 3000 species belonging to the following eight phyla: Actinomycetota, Bacillota, Bacteroidota, Campylobacterota, Fusobacteriota, Pseudomonadota, Thermodesulfobacteriota, and Verrucomicrobiota [14,17,18]. Although the taxonomic and functional composition of the GM is shaped by several host-related and environmental factors (e.g., genetics, age, dietary habits, drug consumption, psychological stressors) [19,20,21,22,23,24], certain bacterial genera consistently dominate in healthy individuals including *Lactobacillus*, *Bacillus*, *Clostridium*, *Enterococcus*, *Ruminococcus*, *Faecalibacterium*, *Roseburia*, *Blautia*, *Dorea*, and *Eubacterium* (phylum Bacillota); *Bacteroides* and *Prevotella* (phylum Bacteroidota); *Bifidobacterium* (phylum Actinomycetota); and *Escherichia* (phylum Pseudomonadota) [14,16,25]. However, disruption of the ecological balance of the GM leads to a state known as bacterial dysbiosis, which affects microbial diversity, alters metabolic and immune-related functions, and compromises intestinal barrier integrity [26].

A substantial body of evidence has revealed the existence of a connection between the brain and the gut via the vagus nerve and chemical molecules including microbial metabolites, hormones, and neurotransmitters [12,27,28]. In particular, specific gut microorganisms and their metabolic by-products have been identified as key regulators of host physiology, contributing to the modulation of the immune system [29,30], inflammatory processes [31,32], and brain function and behavior [33,34]. These findings have progressively highlighted the GM as a potential modulator of the neurobiological mechanisms involved in SUDs. However, despite the considerable number of studies investigating the link between the GM and SUDs in animal models, research in humans remains scarce and has primarily focused on the effects of individual substances [12,35,36]. In response, this narrative review provides an updated overview of how drugs of abuse influence the composition and dynamics of the human GM and how bacterial dysbiosis may be a contributing factor to SUDs by modulating the communication between the gut and the brain. Thus, by examining commonly abused substances such as alcohol, psychostimulants, opioids, cannabinoids, and nicotine, this review aims to deepen the understanding of the bidirectional relationship between the GM and substance use.

## 2. Method

The present review adopted a narrative approach, synthesizing the existing literature in a qualitative and interpretative manner to provide an overview of the topic and present an informed analysis [37]. Both authors independently conducted an extensive literature search aligned with the subject under investigation. For this purpose, the PubMed, Scopus, and Web of Science databases were examined from January to February 2025 using various combinations of terms related to the research topic. The search strategy also involved reviewing reference lists from previous studies and research articles. All relevant records were independently evaluated by both authors, considering studies conducted on humans and focusing on the impact of substances of abuse on the GM or vice versa. In the initial stage, the title and abstract of each article were reviewed for relevance. Duplicate entries were removed as well as studies unlikely to meet the inclusion criteria due to their subject matter. The remaining articles were thoroughly assessed, and pertinent data were extracted for further analysis. Studies lacking substantial information on the relationship between the human GM and substance use or SUDs as well as those based on meta-analyses of endocrine disorders or related to physiological, autoimmune, or viral diseases were excluded from the review.

## 3. Substances of Abuse and Human GM Composition

Recent interest has focused on the potential role of GM dysbiosis in the pathogenesis of SUDs, with some evidence suggesting that GM alterations may influence addiction via changes in gut-brain signaling. Furthermore, changes in the GM and its metabolites may not only result from SUDs, but could also modulate behavioral responses to substances of abuse. The following section examines the impact of various substances on GM composition.

### 3.1. Alcohol

Alcohol is one of the most ancient and widely consumed psychoactive substances, primarily acting as a central nervous system depressant by enhancing γ-aminobutyric acid (GABA) activity, the brain’s main inhibitory neurotransmitter, thereby exerting sedative and anxiolytic effects [38]. It also targets N-methyl-D-aspartate (NMDA) receptors, which are key components of the glutamatergic system involved in synaptic plasticity, learning, and memory, further contributing to its neurodepressive action [39]. Chronic alcohol misuse leads to alcohol use disorder (AUD), a complex condition that encompasses a broad spectrum of neurological, metabolic, and psychological symptoms, with significant implications for individual, social, and public health [40]. Notably, severe withdrawal syndrome related to AUD (i.e., *delirium tremens*) is associated with high mortality rates [41]. In terms of immune function and disease, alcohol displays a dose-dependent relationship: high levels of consumption are consistently associated with increased risk for both infectious and non-communicable diseases, whereas low-to-moderate intake may confer protective effects in specific contexts including certain autoimmune diseases [42,43]. Nevertheless, the classification of drinking patterns remains inconsistent across the literature, with substantial variability in the definitions of low, moderate, and heavy alcohol consumption [44]. In addition, large interindividual and interethnic differences in alcohol-induced toxicity have been attributed to genetic and environmental variability in ethanol metabolism [45]. Moreover, recent studies have identified a direct link between alterations in GM composition and both acute and chronic alcohol consumption, highlighting a potential pathway through which this substance exerts systemic effects [46,47].

Various studies have indicated that alcohol consumption produces an increase in the abundance of the bacterial families *Erysipelotrichaceae, Enterobacteriaceae*, and *Lachnospiraceae,* in the genera *Bacteroides*, *Sutterella*, *Streptococcus, Holdemania,* and *Clostridium* as well as a decrease in the genera *Akkermansia* and *Faecalibacterium* [48,49,50,51]. More recently, Du et al. [52] reported alterations in GM diversity and composition among patients with AUD, characterized by reduced α-diversity and elevated β-diversity indices. These dysregulated indices included a lower abundance of members from the genera *Bacteroides*, *Faecalibacterium, Dialister*, *Clostridium* cluster XIVa, *Lachnospiraceae incertae sedis*, and *Gemmiger*, alongside the increased representation of genera including *Prevotella, Megamonas, Escherichia*, *Coprobacillus*, *Clostridium, Gemella, Rothia*, and *Fusobacterium*. In patients with alcoholic cirrhosis, Baltazar-Díaz et al. [53] found an increase in *Escherichia/Shigella* and *Prevotella* and a decrease in the *Blautia* and *Faecalibacterium* genera.

Dysbiosis associated with alcohol consumption has been shown to provoke a series of biochemical, physiological, and immunological alterations including oxidative stress and the downregulation of antibacterial peptides such as α-defensins [54,55]. Furthermore, microbial dysbiosis has been demonstrated to compromise the integrity of the intestinal mucous barrier by increasing circulating pro-inflammatory cytokines like tumor necrosis factor (TNF-α) and interleukin IL-1β [56,57]. This phenomenon may, in turn, contribute to the development of liver diseases including alcoholic hepatitis and chronic alcohol-related cirrhosis [55,56,58].

The augmented prevalence of Bacteroidota and Fusobacteriota in AUD patients suggests a potential implication of alcohol consumption in colorectal cancer pathogenesis, given the established correlation between alcohol metabolism and the subsequent tumorigenesis driven by *Fusobacterium* [59]. Conversely, the heightened prevalence of Pseudomonadota members, including *Escherichia* and *Shigella* genera, in individuals with AUD [48] could be associated with gut inflammation [60]. This inflammation has been shown to trigger IL-1β and corticosterone production, which has been related to depression and cognitive impairment [61,62]. Moreover, the levels of *Faecalibacterium* were reduced in AUD individuals, indicating that alcohol intake results in negative outcomes for beneficial gut bacteria, which plays a pivotal role on anticancer immunosurveillance and liver pathologies [63,64].

The variation in the abundance of *Faecalibacterium*, *Gemmiger*, *Escherichia*, and *Fusobacterium* in the GM of AUD individuals has been suggested as an indicator to predict cognitive impairment in domains such as emotional processing, memory, and executive functions [65,66]. Indeed, numerous authors have underscored the pivotal role of the GM in emotional and social cognition including drug addiction [15,67,68]. In this sense, Ling et al. [69] reported that the abundance of *Faecalibacterium* and *Gemmiger*, both butyrate-producing genera, was reduced in AUD patients, which positively correlated with cognitive functions [70], while showed a negative correlation with inflammatory cytokines like TNF-α and chemokines [69].

#### Binge Drinking and Alcohol Craving

The term “binge drinking” defines the rapid consumption of a substantial quantity of alcohol within a short timeframe, leading to a sudden increase in blood alcohol concentration [71]. This practice is particularly prevalent during adolescence, a period when social and emotional traits are undergoing significant development [67,72,73,74,75]. Consequently, binge drinking may imply a significant impact on future alcohol consumption patterns [76]. Carbia et al. [77] examined the relationship between binge drinking and GM dysbiosis, along with the concomitant social cognition. Their findings revealed that among adolescents engaging in binge drinking, there was no difference in α-diversity index. On the other hand, β-diversity exhibited a relationship with alcohol consumption, with changes in diversity contingent on the amount of alcohol consumed. The GM composition showed significant alterations in the genera *Alistipes* (decreases) and *Veillonella* (increases), although binge drinking was also linked to changes in the abundance of *Bacteroides* spp., *Blautia wexlerae*, *Ruminococcus lactaris,* and *Coprococcus eutactus*. Concerning this issue, β-diversity differences associated with young people and the relationship between decreases in *Alistipes* or increases in *Veillonella* with several liver diseases have been reported by different authors [78,79,80,81].

Alcohol craving, a critical mechanism implicated in AUD, is characterized by an intense urge or irrepressible desire to consume alcohol. Impulsivity has been noted as a pivotal factor in the development of craving [82]. Nevertheless, associations with inhibition and thought suppression continue to be insufficiently clarified in the current body of research [83]. Alcohol craving has been associated with a decrease in *Ruthenibacterium lactatiformans* (family *Ruminococcaceae*), and a reduced abundance of members of this taxon has also been related to high levels of craving in AUD patients [68]. In the context of social cognition, Carbia et al. [77] observed that a decline in the levels of *Clostridium* spp., *Flavonifractor plautii*, and *Eggerthella lenta*, accompanied by an increase in *Coprococcus* spp., was linked to a diminished recognition of sadness. Conversely, heightened impulsivity was related to a decrease in *Collinsella* spp. and to an increase in *Roseburia* and *Parabacteroides* spp.

### 3.2. Psychostimulants

Psychostimulants are substances that indirectly activate the sympathetic nervous system, sharing characteristics with sympathomimetic agents and possessing a significant risk for abuse and dependency. They primarily act by increasing synaptic dopamine release and enhancing the availability of monoamines like dopamine, norepinephrine, and serotonin [84]. Psychostimulants are well-known for their energizing effects and widespread use, encompassing both prescribed and non-prescribed applications, which is why they are commonly consumed to enhance performance and for recreational purposes [85]. Psychostimulants exhibit diverse chemical structures and mechanisms of action, contributing to their varying effects on neurotransmitter systems, which result in differences in mood, alertness, and cognitive performance [86]. Prolonged use may lead to SUDs, manifesting as clinically significant impairments and distress [87]. Psychostimulants can be classified into two major groups: those that act as passive monoamine reuptake inhibitors (e.g., cocaine) and those that stimulate monoamine release by serving as substrates for monoamine transporters (e.g., amphetamines). Despite differences in their chemical structures, both classes produce similar discriminative stimulus effects, primarily mediated by dopaminergic mechanisms, although these effects can also be modulated by changes in the noradrenergic and serotonergic systems [86]. The abuse of psychostimulants induces various neurochemical changes, including alterations in dopaminergic, glutamatergic, serotonergic, and GABAergic signaling, and is associated with oxidative stress and epigenetic modifications, such as altered DNA methylation and gene expression, contributing to neurological impairments [88,89]. Although clinical studies on the influence of psychostimulants on the GM are limited, emerging evidence points to a bidirectional relationship between psychostimulant use and GM composition, with the GM modulating the behavioral response of the brain and vice versa [84].

Clinical studies on the influence of psychostimulants (e.g., cocaine, amphetamines, methamphetamine, MDMA) on the human GM are sparse. However, research has revealed a reciprocal relationship between the GM and psychostimulant drugs, with the latter modulating the brain’s behavioral response [84]. In a study on cocaine users, Volpe et al. [90] reported that drug use was linked to an increased and decreased abundance in the Bacteroidota and Bacillota phyla members, respectively. However, a study on methamphetamine use revealed no significant alterations in GM diversity [91], although the drug administration was associated with increases in *Murdochiella* and *Eubacterium*, along with a decrease in *Butyricicoccus* and *Faecalibacterium*. Concurrently, other clinical studies have demonstrated that methamphetamines compromise the intestinal barrier and trigger inflammatory responses, thereby disrupting the gut ecosystem [92,93].

Yang et al. [94] reported significant reductions in Deltaproteobacteria and *Bacteroidaceae* levels, alongside an increased abundance of Xanthomonadales, Sphingomonadales, *Lachnospiraceae*, and *Romboutsia* in the GM of methamphetamine users. Moreover, the abundance of Fusobacteriota phylum was found to be correlated with the duration of drug use. In a recent study performed by He et al. [95], it was established that participants with methamphetamine use disorder (MUD) and methamphetamine causal use (MCU) exhibited significant changes in their GM composition compared with the control group. The most prevalent phyla in the MCU and MUD groups were Bacillota (70%), Bacteriodota (20%), Pseudomonadota (5%) and Actinomycetota (>2%). At the genera level, the GM showed a preponderance of *Faecalibacterium*, *Bacteroides*, *Roseburia*, *Ruminococcus*, *Megamonas*, *Prevotella*, *Lachnospira*, *Blautia*, *Coprococcus*, and *Dialister* among both groups. These authors also reported higher abundance of the *Clostridiaceae, Halomonadaceae*, *Hyphomicrobiaceae*, and *Xanthomonadaceae* families, and the *Halomonas*, *Clostridium*, *Devosia*, and *Dorea* genera in the GM of the MUD group compared with the MCU group. In the context of cocaine, Gerace et al. [96] observed that the predominant genera in stool samples of consumers were *Bacteroides*, *Bifidobacterium*, *Blautia*, *Collinsella*, and *Faecalibacterium*. A subsequent comparison of the GM of cocaine users with that of non-users revealed a significant reduction in the α-diversity among the former group. In addition, they identified higher fecal abundances of *Dorea*, *Erysipelotrichaceae*, *Eubacterium*, *Blautia*, *Collinsella*, *Holdemanella*, *Escherichia/Shigella*, *Megamonas*, *Romboutsia*, *Peptococcus*, *Senegalimassilia*, *Rothia*, *Turicibacter*, and *Streptococcus*. Conversely, a decrease in *Christensenellaceae, Desulfovibrionaceae, Lachnospiraceae, Alistipes*, *Bacteroides*, *Coprobacter*, *Odoribacter*, *Oscillospira*, *Paraprevotella*, *Parasutterella*, *Sutterella*, and *Barnesiella* was found in the cohort of drug users.

GM dysbiosis triggered by psychostimulant use has been linked to a number of physiological diseases including inflammation of the gastrointestinal tract, Crohn’s disease, and colorectal cancer [97,98,99]. Furthermore, the depletion of GM in psychostimulant users has been demonstrated to alter the genes related to synaptic plasticity, resulting in an increased inclination toward drug seeking and use following prolonged abstinence [100,101]. Recent studies have indicated a potential association between genetics and/or neuroimmunology and methamphetamine addiction [93,102,103]. Moreover, methamphetamine-dependent individuals have been observed to exhibit a heightened prevalence of psychotic symptoms and psychiatric disorders [94,95,104]. Anxiety, depression, violent behavior, insomnia, psychosis, and schizophrenia have also been reported in chronic methamphetamine users [98,105,106].

### 3.3. Opioids

Opioids are a group of powerful drugs derived from the opium poppy plant (*Papaver somniferum*), widely used for their potent analgesic and sedative effects [107,108,109]. Several opioids are commonly employed in clinical practice including fentanyl, methadone, morphine, levorphanol, hydromorphone, meperidine, oxymorphone, and oxycodone [110]. Despite their extraordinary clinical utility, opioids carry a high risk of dependency and abuse [111], raising significant concerns regarding their impact on public health including the dramatic recent increase in overdose deaths [112]. Opioids produce their effects by binding to G-protein-coupled opioid receptors (μ, κ, δ, and NOP), which are found in the brain, periphery, and gut [113,114]. These receptors are activated by both endogenous neurotransmitters, hormones, and peptides (e.g., enkephalins, endorphins) as well as exogenous opioids (e.g., heroin, morphine, fentanyl), modulating pain and other physiological processes [114]. However, prolonged opioid use can result in opioid use disorder (OUD), a persistent condition characterized by clinically significant impairment, intense withdrawal symptoms, and recurrent relapse [115]. Moreover, the economic burden of OUD has been reported, affecting insurers, healthcare payers, and individuals or families, contributing to significant financial strain on both personal and public healthcare systems [112]. Opioids also affect the gut by causing peristaltic slowing and constipation, which can lead to changes in gut barrier permeability and promote bacterial translocation [35,115,116,117]. These disruptions contribute to dysbiosis of the GM, which in turn plays a significant role in opioid tolerance. The alterations in GM composition exacerbate the effects of opioids, creating a detrimental positive feedback loop that further impacts gut health and opioid response [118].

The relationship between gut dysbiosis and OUD in humans has received scant attention from the research community [24,112]. The GM of OUD patients has been observed to exhibit an increase in α-diversity, a phenomenon attributed to the time-delay in colon transit induced by the opioids, which has been demonstrated to enhance bacterial proliferation within the gastrointestinal tract [119]. Clinical studies on chronic opioid users have shown a reduction in members of the phylum Bacteroidota, family *Bacteroidaceae*, and genus *Bacteroides* [24,120]. Contradictory results have also been reported regarding the abundance of the genera *Prevotella, Bifidobacterium*, and *Ruminococcus* as well as of the family *Ruminococcaceae* in the GM of individuals with OUD [24,120,121]. In turn, opioid use also induces other GM changes, exerting a deleterious effect on members of the family *Bacteroidaceae* and the genera *Lactobacillus* and *Bifidobacterium* [112]. Concurrently, it has been demonstrated to stimulate an increase in the prevalence of several pathogenic genera such as *Enterococcus*, *Flavobacterium*, *Fusobacterium*, *Sutterella*, *Ruminococcus*, and *Clostridium* [122,123]. Interestingly, *Bacteroidaceae* members have been shown to prevent the overgrowth of these nosocomial pathogenic bacteria, which are a cause of serious microbial infections that necessitate hospitalization [124]. The association of opioid use with an elevated risk of sepsis has been posited by several authors [115,125,126].

Numerous studies have documented the implications of OUD on diverse immune system functions, such as the upregulation of pro-inflammatory cytokines following short- or prolonged-exposure to opioids, the enhanced activation of microglia, the attenuation of the immune response to microbial pathogens [84,127,128], and the disruption of intestinal permeability, resulting in systemic bacterial propagation [125,129]. The neuroinflammation that results from prolonged opioid use may play a role in the development of drug-related symptoms including tolerance, dependence, reward processing, anxiety, and depression [84,122,130].

### 3.4. Cannabinoids

Cannabinoids are bioactive compounds found in the cannabis plant (*Cannabis sativa*) that interact with the endocannabinoid system (ECS), including psychoactive Δ-9-tetrahydrocannabinol (Δ^9^-THC) and non-psychoactive compounds such as cannabigerol (CBG), cannabichromene (CBC), and cannabidiol (CBD), among over 560 other identified constituents, all of which are related to natural endocannabinoids (eCBs) [131,132]. Cannabinoids are consumed worldwide for cultural, medicinal, and recreational purposes, exhibiting low dependence potential, mild side effects, and a broad range of therapeutic benefits including anti-inflammatory, antibacterial, and immunosuppressive effects as well as positive outcomes in several physiological, physical, neurological, neurodegenerative, and mental health conditions [133,134,135,136,137,138]. In contrast, cannabis use has also been associated with greater levels of pro-inflammatory cytokines like IL-1β, IL-6, IL-8, and TNF-α [139]. While persistent cannabis use can lead individuals to seek treatment, its impact on health is generally less severe than that of other SUDs [140]. Furthermore, cannabinoids may influence the GM by modulating pain responses and related biological processes through their interaction with the ECS, with THC potentially preventing alterations in microbial composition and CBD supporting gut health and motility, offering a beneficial effect in contrast to the detrimental impact of antibiotics on the microbiota [141,142].

The ECS serves as a crucial link between the brain and the GM, regulating intestinal homeostasis and modulating stress responses [132,143,144,145,146]. The ECS is comprised of two cannabinoid receptors (CB_1_ and CB_2_), their respective endogenous ligands, and the biosynthetic mechanisms and enzymes that regulate the availability of these ligands [147,148]. The psychoactive and gut effects of cannabis are mediated via the CB_1_ and CB_2_ receptors [149]. CB_1_ is expressed in the intestinal epithelium and in the brain. Its activation has been shown to reduce gastrointestinal motility and gastric acid secretion while increasing feeding and binge-like behaviors [150]. CB_2_, in turn, is predominantly expressed in plasmatic cells such as macrophages, with minimal expression in the brain [151]. CB_2_ activation has been implicated in the modulation of intestinal inflammation, the regulation of aberrant gut motility, and the limitation of visceral sensitivity and pain [152].

Considering that the most common method of human consumption of cannabis is via smoking, a paucity of studies has been performed on the effects of this substance on the GM. Cani et al. [143] established that exogenic cannabinoids and the ECS regulate the GM. In a related study, Mehrpouya-Bahrami et al. [153] reported that antagonizing CB_1_ attenuated cytokine release, lowered intestinal permeability, and modified the GM including an increase in *Akkermansia muciniphila* and a decrease in *Lachnospiraceae* and *Erysipelotrichaceae* abundances. In a cohort study involving cannabis users and non-users, Panee et al. [154] noted that the abundance of *Prevotella* and *Bacteroides* exhibited an inverse correlation among participants, with the ratio of *Prevotella* to *Bacteroides* being 13-fold higher in non-users. Human dietary interventions involving specific fatty acids have been shown to elevate the levels of eCBs, which can be attributed to changes in diverse GM such as *Peptostreptococcaceae*, *Veillonellaceae*, and *Akkermansiaceae* [155]. Vijay et al. [156] revealed a positive relationship of ECS with bacterial α-diversity and with short-chain fatty acids (SCFAs)-producers *Bifidobacterium, Coprococcus*, and *Faecalibacterium*, but presented a negative relationship with *Collinsella* and *Escherichia/Shigella*.

### 3.5. Nicotine

Nicotine is a compound derived from the tobacco plant (*Nicotiana tabacum* L.), predominantly consumed through cigarette smoking, consistently used for non-medical purposes, and associated with serious health consequences [157,158,159,160]. It is highly addictive, with dependence developing rapidly, making it difficult to quit [161,162,163]. Nicotine primarily acts as an agonist at most nicotinic acetylcholine receptors (nAChRs), except for the nAChRα9 and nAChRα10 subunits, where it functions as an antagonist [164]. Prolonged exposure to nicotine leads to neuroadaptations, including upregulation and desensitization of nAChRs, while withdrawal symptoms, triggered by the absence of nicotine, are primarily influenced by nAChRs containing α2, α3, α5, and β4 subunits in the epithalamic habenular complex [164]. Nicotine use, whether through smoking or vaping, affects the oral, gut, and respiratory microbiomes [165,166,167]. Smoking is a well-known risk factor for gastrointestinal cancers, Crohn’s disease, and liver disease [168,169,170]. In addition, nicotine negatively impacts specific innate immune cells such as dendritic cells, neutrophils, natural killer cells, and macrophages/monocytes [171]. Nicotine also affects the central nervous system influencing the gut–brain axis and GM composition, with nicotine exposure leading to alterations in bacterial metabolic pathways, neurotransmitter levels, and neuroactive metabolites, which contribute to physiological responses and behavioral outcomes [172].

There is a notable lack of clinical research exploring the impact of tobacco use on the GM. However, smoking withdrawal has been associated with substantial shifts in the GM, including increased microbial diversity and a higher relative abundance of the phyla Bacillota and Actinomycetota, along with a concurrent reduction in members of the Bacteroidota and Pseudomonadota phyla [173]. Subsequent research by Vogtmann et al. [174] examined the impact of smoking on the upper gastrointestinal microbiome. Their findings indicated that smoking was related to increased α- and β-diversities, with the species *Dialister invisus* and *Megasphaera micronuciformis* being the most prevalent in smokers. Conversely, decreases in microbial population diversity as well as in the genera *Faecalibacterium*, *Collinsella*, *Enterorhabdus*, and *Gordonibacter* were observed in smoking Crohn’s disease patients [175]. A parallel observation was made in another study, which reported a decline in *Faecalibacterium* and an increase in *Proteus* levels in smoking hemicolectomy patients when compared with subjects who underwent no surgical resection [176].

Lee et al. [177] reported no differences in α-diversity between three participant groups (never, former, and current smokers). However, the bacterial β-diversity showed a significant difference. Current smokers exhibited an increase in the abundance of the phylum Bacteroidota, and a decrease in Bacillota and Pseudomonadota compared with never smokers, whereas there were no differences observed between former and never smokers. In another study, Savin et al. [167] noted increases in the abundance of the Pseudomonadota and Bacteroidota phyla as well as in the genera *Prevotella*, *Bacteroides*, and *Clostridium*. Furthermore, a decline in the abundance of Actinomycetota and Bacillota phyla, along with the genera *Bifidobacterium* and *Lactococcus*, was also reported by these authors.

Shanahan et al. [178] demonstrated that tobacco smokers exhibited a lower bacterial diversity in the upper small intestinal mucosa in comparison with non-smokers. The GM in smokers presented a higher relative abundance of Bacillota (genera *Streptococcus* and *Veillonella*) and Actinomycetota (genus *Rothia*) and lower levels of Bacteroidota (genus *Prevotella*) and Pseudomonadota (genus *Neisseria*). These findings contrast with those reported by Stewart et al. [179], who observed an increase in Pseudomonadota and Bacteroidota abundance, with the predominant genera being *Clostridium* and *Prevotella*, and a decrease in the genus *Bacteroides*. Moreover, Nolan-Kenney et al. [180] observed a relative increase in the abundance of *Catenibacterium* genus, representatives of the family *Erysipelotrichaceae*, and of the Alphaproteobacteria taxon in current smokers. On the contrary, Lin et al. [181] revealed that cigarette and alcohol use were associated with shifts in the relative abundances of the phyla Bacteroidota and Bacillota, along with changes in over 40 bacterial genera. The most pronounced reduction in relative abundance was detected among members of the *Ruminococcaceae* family. More recently, Antinozzi et al. [182] examined the impact of conventional and electronic (e-) cigarette smoking on the human intestinal microbiota. The analysis showed a substantial increase in the prevalence of the *Prevotella* genus in cigarette smokers, but this increase was not observed in e-cigarette users. Additionally, the study identified a progressive increase in the *Desulfovibrio* genus, depending on the specific type of cigarette consumer, and an augmentation in the Alphaproteobacteria among current-smokers in comparison with non-smokers.

The contradictory results obtained in the aforementioned studies may be explained by the presence of different bacterial species contained in tobacco products [174,183]. In this respect, some of these bacteria are opportunistic potential pathogenic bacteria such as *Campylobacter, Acinetobacter*, *Clostridium*, *Bacillus*, *Burkholderia*, *Pseudomonas aeruginosa*, *Klebsiella, Proteus*, *Serratia, Staphylococcus*, and *Enterococcus* [184]. Another potential explanation relates to the immunosuppressive nature of tobacco, which has been demonstrated to decrease the activity of natural killer cells, thereby increasing the susceptibility to microbial infection [174]. Table 1 presents the primary alterations in GM composition associated with the use of various substances.

## 4. Signal Pathways Between the GM and SUDs

The potential for substances of abuse to influence the GM may have implications for the development of addiction disorders. Although the exact mechanisms by which the GM modulates behavioral responses to drugs of abuse are not fully understood, microbial products such as SCFAs, tryptophan metabolites, bile acids, and neurotransmitters have been suggested to play a role in this process by influencing BBB permeability, host immune activation, neural signaling, and gene expression. The following section examines how these microbial products and their interactions with neural pathways may contribute to the modulation of SUDs.

### 4.1. Intermediate Bacterial Metabolites

SCFAs, such as butyric acid, propionic acid, and acetic acid, are bacterial metabolites that have been linked to a variety of metabolic, immunological, and neural host functions [185,186,187]. SCFAs, along with tyrptophol, an indole derivative, have been shown to regulate host cytokine production. These circulating cytokines have the potential to cross the blood–brain barrier (BBB) and exert their influence on the brain, thereby modulating behavioral response to SUDs [186,188]. In addition, SCFAs have been shown to promote beneficial effects on the host based on their anti-inflammatory properties and on their epigenetic regulation of gene expression [185,189]. Inflammation has been demonstrated to impact glutamatergic signaling, a key neurotransmitter system in drug addiction and relapse [190]. Alterations in peripheral inflammatory processes have the potential to influence drug taking and seeking behaviors in SUDs [84,191,192]. Moreover, SCFAs may act as histone deacetylase inhibitors, stimulate histone acetyltransferases, and serve as molecular substrates for histone post-translational modifications [193,194,195,196]. These modifications are critical for the proper functioning of microglia [197]. While the established role of microglia in immune surveillance is well-defined, emerging evidence suggests a potential complementary role for microglia in regulating behavioral aspects related to SUDs [198]. Furthermore, Walker and Nestler [196] proposed that the mechanisms regulating SUD-associated behaviors could involve changes in gene expression within the mesolimbic dopamine system, which is part of the brain’s reward circuitry.

The GM has been identified as a potential alternative route for the production of kynurenic acid (KYNA), an endogenous tryptophan metabolite [199]. Decreased levels of KYNA, which modulate glutamatergic neurotransmission, have been correlated with alcohol craving in AUD patients [200]. In a similar way, Morales-Puerto et al. [201] reported that the modulation of KYNA metabolism could reduce drug seeking behaviors involving various substances such as alcohol, nicotine, cannabis, amphetamines, cocaine, and opioids.

### 4.2. Signaling Molecules: Bile Acids and Neurotransmitters

Bile acids (BAs) are key signaling molecules that regulate immune homeostasis, inflammation induction, and even cell death. The GM is responsible for the balance of primary and secondary BAs involved in lipid intestinal absorption and in the acquisition of energy [202]. Dysbiosis, which is marked by an imbalance in the composition of the GM, has been observed to reduce the production of secondary BAs, resulting in an over-abundance of primary BAs. This, in turn, has the potential to enhance the bioavailability of drugs to the host or modify drug metabolism [203,204].

The GM produces acetylcholine, serotonin, dopamine, epinephrine, norepinephrine, and GABA, which fulfill crucial functions as neurotransmitters within the CNS [12,205]. Bacterial dysbiosis affects the synthesis and regulation of gut neurotransmitters including serotonin [206]. In a classical study, Ciccocioppo [207] reported that serotonergic 5-hydroxytryptamine (5-HT) plays a role in the regulation of drug intake, and consequently in the maintenance of addictive behaviors. A subsequent study by Müller and Homberg [208] expanded upon this research by conducting a review of the role of 5-HT in behaviors associated with the establishment, transition, and maintenance of addiction to various drugs of abuse including amphetamines, cocaine, methamphetamine, morphine, heroin, MDMA (ecstasy), cannabis, nicotine, and alcohol. These authors identified various drug-specific mechanisms in the 5-HT system including serotonergic adaptations within this system. They also identified genetic risk factors for the establishment of controlled behaviors associated with substance use and for the transition to compulsive substance use behaviors.

The role of dopamine signaling within the nucleus accumbens in relation to the reinforcing effects of drugs is well-established. Moreover, chronic exposure to drugs, a major cause of addiction, has been shown to trigger glutamatergic-mediated neuroadaptations in dopamine striato-thalamo-cortical and limbic pathways including the amygdala and hippocampus [209]. The GM plays an important role in regulating dopamine concentrations within the brain, facilitating its synthesis and modulating its catabolism [210]. Interestingly, GM can metabolize drugs, modifying their effectiveness and pharmacokinetics [211], thereby altering the magnitude of reward and withdrawal symptoms [212].

### 4.3. Neural Pathways

The vagus nerve, which is a part of the parasympathetic nervous system, facilitates indirect bidirectional communication within the brain–gut axis [213]. It receives and responds to signals from gut bacterial metabolites like SCFAs [27,214]. In addition, enteroendocrine cells of the gut epithelium transmit signals to the vagus nerve via the release of serotonin, cholecystokinin, peptide YY, and glucagon-like peptide-1 [27,215]. These interactions between the gut-enteric and the nervous system may potentially influence interoceptive signals that may play a pivotal role in the development of SUDs and other psychiatric conditions [216,217]. While the role of the vagus nerve in SUD behavioral responses is well-documented, the influence of the human GM via the vagal route to modulate these responses remains to be fully elucidated [122].

Microglia constitute resident immune cells in the brain and spinal cord that are mobilized in response to CNS infection or injury and exert protection against many neurodegenerative diseases [19,218]. The activation of microglia has been shown to promote tissue repair and homeostasis through the release of cytokines and the phagocytosis of cellular debris [219]. However, the mechanisms that regulate these functions are not fully understood, especially with regard to the impact of extrinsic factors such as the human GM [220]. The GM, through the secretion of bacterial metabolites and neurotransmitters, modulates the inflammatory response of microglia in the CNS [221]. During SUDs, the resident macrophages of the CNS are activated, and the subsequent TNF-α and IL-1β released by the microglia contribute to the pathophysiology of SUDs [222]. Therefore, the communication between the microglia and GM may be regarded as an important factor in the mechanism of microglial activation during SUDs. This is due to the fact that microglial cells are dependent on a healthy and balanced GM for proper development, maturation, and function. Translocated gut bacteria can release pro-inflammatory cytokines, including TNF-α, IL-1β, and IL-6, which can further cause disruption in the epithelium, affecting the functionality of microglia [223].

Brain-derived neurotrophic factor (BDNF) has emerged as a promising candidate for elucidating the role of the GM in drug addiction. BDNF is synthesized by both neuronal and glial cells as well as by peripheral immune cells and the vascular endothelium [224]. Epigenetic modifications, specifically alterations in histone acetylation, have been identified in the context of cocaine use, and BDNF has been demonstrated to mediate the behavioral effects of opioids [225].

**Figure 1:**
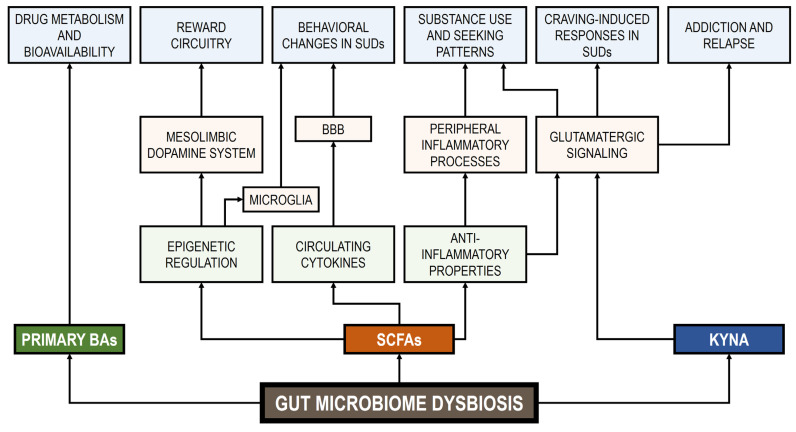
Signal pathways between GM metabolites and SUDs. BBB: blood–brain barrier. BAs: bile acids. SCFAs: short-chain fatty acids. KYNA: kynurenic acid.

The hypothalamic–pituitary–adrenal (HPA) axis, a neuroendocrine system linked to stress-related processes, is responsible for cortisol production in humans. Appropriate levels of this glucocorticoid are essential for normal neurodevelopment and neural function, and it also participates in several cognitive processes such as learning and memory [226]. Evidence suggests an intricate interconnection between the GM, the HPA axis, and cognitive processes through various substances (e.g., neurotransmitters, hormones, bacterial metabolites) and pathways (e.g., vagus nerve, immune system, and BBB regulation) [227]. Gut dysbiosis, resulting from drug use and other factors, can impact the stress response of the host, HPA axis activity, and even cognitive health [227]. The impact of stress on drug craving and consumption has been a subject of considerable research, with findings indicating that stress hormones may play a role in regulating these behaviors [228]. However, the specific mechanisms through which stress hormones influence addiction memory remain to be fully elucidated. Figure 1 shows various potential signal pathways between GM metabolites and SUDs.

## 5. SUD Treatments

The treatment of SUDs presents significant challenges due to their complex and multifactorial etiology as well as the wide variety of effects associated with different substances of abuse [1]. For instance, treatments for alcohol, tobacco, and opioid use disorders frequently prove ineffective or unsuitable [229,230,231], while treatments for other substances such as cannabis or psychostimulant use disorders are not yet approved [232,233,234].

Current therapeutic interventions for SUDs are multimodal, encompassing and including psychotherapy, behavior modification, and pharmacotherapy [235]. However, the variability in the efficacy of these treatments has led to the proposal of alternative approaches including support group treatment, neuromodulation, and GM interventions [236,237,238,239]. A substantial body of evidence from animal models supports the notion that the GM could be a viable therapeutic target for various SUDs, with probiotics, prebiotics, and fecal microbiota transplantation (FMT) being the main approaches [192,213]. In clinical studies, the microbial treatment of alcohol detoxification patients using *Bifidobacterium bifidum* and *Lactiplantibacillus* (formerly *Lactobacillus*) *plantarum* has shown beneficial outcomes by lowering liver enzymes [240]. Conversely, patients diagnosed with AUD who exhibited GM dysbiosis presented an augmentation in the proportion of *Faecalibacterium prausnitzii* and *Bifidobacterium* spp. following the administration of prebiotics derived from galactooligosaccharide or inulin-type fructans [241]. In a study by Bajaj et al. [242], an FMT procedure to enrich *Lachnospiraceae* and *Ruminococcaceae* was performed on subjects diagnosed with AUD and suffering cirrhosis. The subjects who underwent the intervention exhibited a reduction in alcohol cravings. In a more recent study, Letchumanan et al. [238] reported that probiotics, specifically *Lactobacillus* spp. and *Bifidobacterium* spp., provided promising results in reducing endotoxemia and systemic inflammation, thereby protecting against alcohol-induced neuroinflammation. Interestingly, prebiotic treatment based on inulin supplemented with dietary fiber during alcohol withdrawal has been shown to modulate the intestinal microbiota, increase the serum levels of BDNF, and improve social behavior in patients with AUD [243].

In an alternative approach, Lee et al. [244] employed an opioid antagonist (naltrexone), which impedes the binding of opioid agonists to the μ-opioid receptor, to prevent opioid relapse. This opioid antagonist has also been used to manage cravings associated with both AUD and OUD [245], alleviate opioid-induced constipation [246], and reduce gut mucosal injury associated with Crohn’s disease [247]. Treatment with the opioid agonist buprenorphine-naloxone was found to reverse the respiratory depression that occurs during an opioid overdose [244]. While these findings were not conclusive, both treatments (antagonist and agonist) were equally safe and effective in controlling the overall opioid relapse. A separate study has indicated that the utilization of opioid agonists is linked to a reduction in GM diversity, as evidenced by a decrease in the relative abundances of butyrate-producing *Roseburia* and of BA-metabolizing *Bilophila* genera [248].

## 6. Discussion

**Figure 2:**
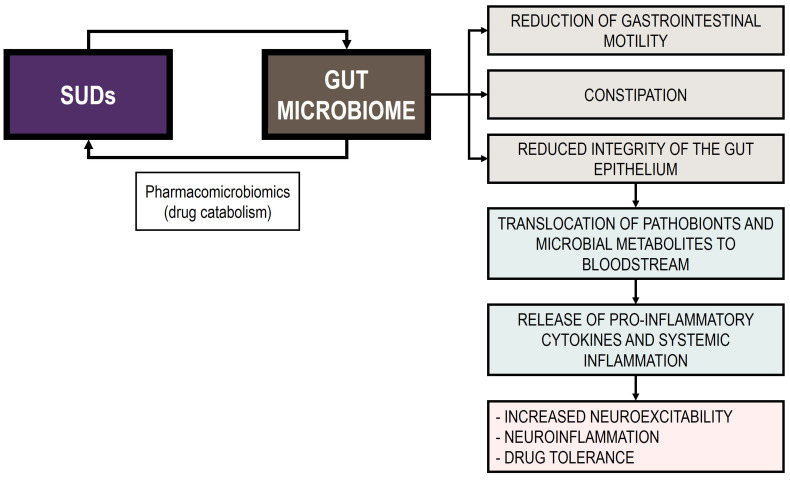
Bidirectional interplay between substances of abuse and the GM.

In this review, we summarize the current evidence on the bidirectional interaction between substance of abuse consumption and GM homeostasis, underscoring its role in the development of SUDs. Indeed, this complex interplay appears to profoundly affect both gut and brain health [249]. In this regard, evidence suggests that substance abuse induces gut dysbiosis, characterized by changes in bacterial diversity, disrupted GM composition, and reduced SCFA levels [35]. In addition, the examination of the pathogenetic mechanisms revealed that drug-induced dysbiosis could be linked to compromised gut permeability and heightened local and systemic inflammatory response. These shifts trigger a cascade of physiological and behavioral responses that exacerbate SUDs and reinforce drug dependence. Figure 2 presents the bidirectional interplay between substances of abuse and the GM.

Experimental research has demonstrated the substantial impact of the GM on SUDs, acting via mechanisms such as altered gene expression in the nucleus accumbens [250], the modulation of reward and addiction circuits [251], and changes in pain perception [252,253]. These results reinforce the direct involvement of the GM in both reward-related responses to substances of abuse [254] and withdrawal states [209], highlighting its relevance across multiple phases of addiction. Within this compelling physiological framework, targeting the GM has emerged as a promising strategy to counteract the detrimental effects of SUDs and enhance compliance with treatment interventions.

Despite the established relationship between the GM and substances of abuse, several potential limitations must be considered in studies examining this association. These include: (i) confounding variables, as many studies have failed to account for pre-existing mental health conditions; (ii) limited sample diversity, with some studies restricted in terms of age or cultural background, making it difficult to generalize findings across different demographic groups; (iii) the cross-sectional design of many studies, which limits their ability to establish causal relationships; (iv) self-reporting bias; (v) the influence of genetic and environmental factors; (vi) lack of long-term follow-up; (vii) the varying effects of SUDs across developmental stages; and (viii) substance use co-occurrence, which may lead to inconsistencies in the findings.

Emerging evidence highlights the significance of the GM in the pathogenesis of SUDs in animal models. However, further research is necessary to substantiate this association in human populations. In the future, it will be desirable to design studies aimed at identifying the different factors involved in this relationship such as the impact of drug exposure duration on the human host microbiome and how changes in GM composition influence SUD symptoms. While recent inquiries have explored whether the GM constitutes a potential environmental risk factor or a potential indicator of SUDs, current clinical studies have yet to provide definitive answers. Research on the GM and SUDs should also prioritize more diverse, representative populations and long-term, longitudinal designs. Incorporating objective biomarkers and self-reports as well as accounting for genetic and environmental variables would help mitigate biases and improve the reliability of the findings. Moreover, exploring the impact of substance use across different developmental stages could lead to more precise and customized interventions. In addition to methodological issues, it is pivotal to reassess the dominant narrative surrounding substances of abuse. In this sense, the consumption of these substances is often stigmatized, lacking a comprehensive understanding, which in turn tends to obscure open discussion and hinder the development of accurate knowledge, particularly among adolescents. In fact, this may inadvertently fuel curiosity and increase the likelihood of experimentation within young populations [1], potentially resulting in reckless patterns of consumption that exceed reasonable limits [255]. Thus, viewing substance use through a lens of condemnation fails to acknowledge the complexity of the issue and may contribute to negative outcomes such as risky behaviors and the reinforcement of stigma toward users. A more balanced, scientifically informed perspective would not only reduce stigma, but also provide a clearer understanding of the physiological and psychological impacts of substance use, individual susceptibility, and how these insights can inform more effective prevention and treatment strategies as well as the creation of optimal environments.

It is essential to recognize that many psychoactive substances, when used in non-abusive settings, confer therapeutic benefits. For instance, opioids are indispensable tools in clinical practice for anesthesia and pain management [112], moderate alcohol consumption has been linked to improved cardiovascular health and antioxidant effects [256], cannabis has demonstrated effectiveness across a wide range of pathological conditions including its key role as an antiemetic [257], and ecstasy has shown potential applications in the treatment of various psychiatric disorders [258]. Nevertheless, these substances can also impact the GM, even with short-term use. Therefore, a promising direction for future research would be to investigate the effects of such substances on the GM under non-abusive conditions. To date, a paucity of research has examined GM alterations following low-dose or short-term exposure. In the case of alcohol, Lee et al. [259] reported that short-term, low-dose alcohol consumption in mice resulted in a decreased abundance of members of the phylum Bacteroidota and an increased abundance of Bacillota. Specifically, *Muribaculum intestinale* was identified as the predominant species and a key contributor to the phylum-level shifts induced by alcohol. The prevalence of *M. intestinale* accounted for over 60% of the total in the control group, but its abundance was significantly reduced after just one week of alcohol intake. Similarly, a growing body of evidence supports the effectiveness of psychostimulants in alleviating symptoms of inattention and hyperactivity/impulsivity in individuals with attention-deficit/hyperactivity disorder (ADHD). Boonchooduang et al. [260] investigated the effects of psychostimulant medication on GM composition and SCFA levels. They found that medicated children with ADHD exhibited significantly reduced microbial diversity and lower abundances of several bacterial taxa, including *Ruminococcaceae*, *Haemophilus*, *Enhydrobacter*, *Kytococcus*, *Micrococcus*, *Staphylococcus*, *Corynebacterium*, *Brevundimonas*, and *Odoribacter*, compared with their untreated counterparts. In contrast, *Anaerostipes* was more abundant in the medicated group, and *Parvimonas* was significantly more prevalent in medicated children compared with healthy controls. In the context of therapeutic cannabis use, various clinical and preclinical studies have investigated its effects on GM composition. In clinical research, Vijay et al. [156] reported a positive correlation between eCBs and bacterial α-diversity as well as with SCFA-producing genera such as *Bifidobacterium*, *Coprococcus*, and *Faecalibacterium*. In a study examining the impact of drug use on GM during HIV infection, Fulcher et al. [261] found that cannabis consumption was associated with an increased abundance of the genera *Clostridium*, *Fusobacterium*, *Ruminococcus*, and *Solobacterium* and a decreased abundance of *Acidaminococcus*, *Anaerostipes*, *Dialister*, *Dorea*, and *Prevotella*. Similarly, in a cohort comparing cannabis users and non-users, Panee et al. [154] observed a positive correlation between cannabis use and *Prevotella* abundance and an inverse correlation with *Bacteroides*. Notably, the *Prevotella*/*Bacteroides* ratio was found to be 13 times higher in non-users. Preclinical studies also revealed substantial alterations in GM associated with cannabis exposure. Mehrpouya-Bahrami et al. [153] observed that cannabis administration in mice led to increased levels of *A. muciniphila* and a decreased abundance of *Lachnospiraceae* and *Erysipelotrichaceae*. Furthermore, THC administration was shown to enhance the abundance of the beneficial species *Ruminococcus gnavus* while reducing the levels of the potentially pathogenic *A. muciniphila* in both the lung and gut [262]. The therapeutic use of opioids has long raised concerns, particularly regarding their impact on the GM. However, only a limited number of studies have explored this effect under medically supervised conditions. Wang et al. [123] demonstrated that morphine administration promoted the growth of pathogenic bacterial communities and that *Enterococcus faecalis* contributed to morphine-induced analgesic tolerance in mice. Supporting these findings, Zhang et al. [263] provided direct evidence of morphine-induced alterations in the rat GM. The authors suggested that a baseline reduction in *Olsenella* and *Rothia*, alongside an increase in *Helicobacter*, may serve as a potential biomarker for heightened vulnerability to addictive behaviors following morphine exposure.

In the context of this discussion, it is worth mentioning the utilization of probiotics as a promising adjunct therapy for SUDs, either as a standalone intervention or as a pharmacotherapy supplement. Preclinical studies have reported the potential benefits of probiotic supplementation in relation to drug abuse. For instance, administration of the commercial probiotic mixture VSL#3, which contains the bacterial species *Lactobacillus acidophilus*, *Lactobacillus delbrueckii* subsp. *bulgaricus*, *Lactiplantibacillus plantarum*, *Lacticaseibacillus casei*, *Bifidobacterium breve*, *B. longum*, *B. longum* subsp. *infantis*, and *Streptococcus salivarius* subsp. *thermophilus*, mitigated the development of analgesic tolerance to morphine in mice. This effect was associated with a partial restoration of GM components and a reduction in proinflammatory cytokines [264]. In addition, Ezquer et al. [265] noted that combined administration of the probiotic strain GG of *Lacticaseibacillus rhamnosus* with N-acetylcysteine and acetylsalicylic acid modulated dopaminergic activity and reduced relapse to ethanol consumption, highlighting its potential as a probiotic adjuvant in AUD treatment. However, further research is needed to optimize probiotic interventions (e.g., combination of probiotic strains and dosage) in clinical studies targeting SUDs. Within this framework, Sarkar et al. [213] proposed the category of psychobiotics including probiotics, prebiotics, or both in combination (synbiotics), which impact microbial-neural signaling. Psychobiotics have demonstrated significant benefits in addressing various psychological adverse states, including stress, anxiety, and depression [266], which could contribute to the alleviation of some of the aversive symptoms associated with drug use such as cravings or withdrawal. Therefore, a promise area for future investigation will involve clinical trials evaluating the effects of psychobiotics on drug addiction. Moreover, the emerging field of nutritional psychiatry could play a pivotal role, emphasizing the importance of diet and gut health in modulating psychological well-being and supporting addiction treatment strategies [267].

Finally, concerted efforts to conceptualize and operationalize randomized controlled trials in human subjects are imperative to ascertain the microbial features associated with SUDs. In addition, therapies that target novel pathways implicated in microbial signaling within the brain–gut axis will enhance our understanding of the role of the GM in drug addiction.

## 7. Conclusions

A substantial body of research, including both preclinical and human studies, has demonstrated a bidirectional relationship between the GM and SUDs. This relationship encompasses the impact of SUDs on GM composition and dynamics as well as the role of GM dysbiosis in mediating behavioral responses to substances of abuse. As a result, manipulating the GM or its by-products may represent a promising approach for SUD treatments. Another important aspect that merits consideration is the potential use of human microbiomes (e.g., oral, gut, upper respiratory tract) or their bacterial metabolites in identifying individuals at increased risk of pathological drug use. Furthermore, novel insights into the interactions between host genetics and the GM may be pivotal for elucidating the role of the microbiome in SUDs and addiction behaviors.

## Figures and Tables

**Table 1:** Influence of various substances on the human GM composition.

Phyla, Families, and Genera	ALC	BIN	CRA	COC	MET	OPI	CAN	NIC
ACTINOMYCETOTA								
*Collinsella*				OOO		OOO		OOO
*Rothia*	OOO			OOO				OOO
*Bifidobacterium*				OOO		OOO		OOO
*Senegalimassilia*				OOO				
*Enterorhabdus*								OOO
*Gordonibacter*								OOO
BACILLOTA								
*Erysipelotrichaceae*	OOO			OOO			OOO	OOO
*Lachnospiraceae*	OOO			OOO	OOO		OOO	
*Ruminococcaceae*			OOO			OOO		OOO
*Christensenellaceae*				OOO				
*Peptostreptococcaceae*							OOO	
*Clostridiaceae*					OOO			
*Holdemania*	OOO							
*Clostridium*	OOO				OOO	OOO		OOO
*Faecalibacterium*	OOO			OOO	OOO		OOO	OOO
*Ruthenibacterium*			OOO					
*Streptococcus*	OOO			OOO				OOO
*Gemmiger*	OOO							
*Dialister*	OOO				OOO			OOO
*Megamonas*	OOO			OOO	OOO			
*Blautia*	OOO	OOO		OOO	OOO			
*Coprococcus*		OOO			OOO		OOO	
*Roseburia*					OOO			
*Ruminococcus*		OOO			OOO	OOO		
*Gemella*	OOO							
*Coprobacillus*	OOO							
*Veillonella*		OOO					OOO	OOO
*Dorea*				OOO	OOO			
*Eubacterium*				OOO	OOO			
*Butyricicoccus*					OOO			
*Romboutsia*				OOO	OOO			
*Lachnospira*					OOO			
*Holdemanella*				OOO				
*Peptococcus*				OOO				
*Turicibacter*				OOO				
*Oscillospira*				OOO				
*Enterococcus*						OOO		
*Lactococcus*								OOO
*Catenibacterium*								OOO
*Megasphaera*								OOO
BACTEROIDOTA								
*Bacteroidaceae*					OOO	OOO		
*Bacteroides*	OOO	OOO		OOO	OOO	OOO	OOO	OOO
*Prevotella*	OOO				OOO	OOO	OOO	OOO
*Alistipes*		OOO		OOO				
*Coprobacter*				OOO				
*Odoribacter*				OOO				
*Paraprevotella*				OOO				
*Barnesiella*				OOO				
*Flavobacterium*						OOO		
FUSOBACTERIODOTA								
*Fusobacterium*	OOO					OOO		
PSEUDOMONADOTA								
*Enterobacteriaceae*	OOO							
*Halomonadaceae*					OOO			
*Hyphomicrobiaceae*					OOO			
*Xanthomonadaceae*					OOO			
*Escherichia*	OOO			OOO		OOO		
*Sutterella*	OOO			OOO		OOO		
*Halomonas*					OOO			
*Devosia*					OOO			
*Parasutterella*				OOO				
*Neisseria*								OOO
VERRUCOMICROBIOTA								
*Akkermansia*	OOO						OOO	
THERMODESULFOBACTERIOTA								
*Desulfovibrionaceae*				OOO				
*Desulfovibrio*								OOO

Rectangles in green: increase. Rectangles in red: decrease. Rectangles in blue: Contradictory results. ALC: alcohol use. BIN: binge drinking. CRA: alcohol craving. COC: cocaine use. MET: methamphetamine use. OPI: opioid use. CAN: cannabinoid use. NIC: nicotine use.

## Data Availability

No new data were created or analyzed in this study. Data sharing is not applicable to this article.

## Abbreviations

The following abbreviations are used in this manuscript:

AUD	Alcohol use disorder
BAs	Bile acids
BBB	Blood–brain barrier
BIN	Binge-drinking
BDNF	Brain-derived neurotrophic factor
CAN	Cannabinoid use
CRA	Alcohol craving
CUD	Cocaine use disorder
ECS	Endocannabinoid system
FMT	Fecal microbiota transplantation
GABA	γ-Aminobutyric acid
GM	Gut microbiome
HPA	Hypothalamic-pituitary-adrenal
5-HT	5-Hydroxytryptamine
KYNA	Kynurenic acid
MUD	Methamphetamine use disorder
NIC	Nicotine use
OUD	Opioid use disorder
SCFAs	Short-chain fatty acids
SUDs	Substance use disorders
TNF-α	Tumor necrosis factor-alpha
